# Survival Is Worse in Patients Completing Immunotherapy Prior to SBRT/SRS Compared to Those Receiving It Concurrently or After

**DOI:** 10.3389/fonc.2022.785350

**Published:** 2022-05-27

**Authors:** Susan Woody, Aparna Hegde, Hyder Arastu, M. Sean Peach, Nitika Sharma, Paul Walker, Andrew W. Ju

**Affiliations:** ^1^ Department of Radiation Oncology, Brody School of Medicine at East Carolina University, Greenville, NC, United States; ^2^ Department of Hematology and Oncology, University of Alabama at Birmingham School of Medicine, Birmingham, AL, United States; ^3^ Anne Arundel Medical Center’s DeCesaris Cancer Institute, Annapolis Oncology Center, Annapolis, MD, United States; ^4^ Brody School of Medicine at East Carolina University, Greenville, NC, United States; ^5^ Circulogene, Birmingham, AL, United States

**Keywords:** SBRT, SRS, immunotherapy, abscopal effect, radiation

## Abstract

**Purpose/Objectives:**

The abscopal effect could theoretically be potentiated when combined with immunomodulating drugs through increased antigen production. The optimal dosing and schedule of radiotherapy with immunotherapy are unknown, although they are actively investigated in laboratory and clinical models. Clinical data in patients treated for metastatic disease with both modalities may guide future studies.

**Materials and Methods:**

This is a single-institution retrospective review of all patients treated with stereotactic body radiotherapy (SBRT)/stereotactic radiosurgery (SRS) and immunomodulating therapy within 6 months before or after SBRT/SRS for metastatic cancer. Clinical and tumor characteristics were recorded, as well as SBRT/SRS details, immunotherapy details, and survival. Log-rank tests on Kaplan–Meier curves for overall survival (OS) that were calculated from the end of SBRT/SRS were used in univariate analysis and Cox proportional hazards regression for multivariate analysis.

**Results:**

A total of 125 patients were identified who met the inclusion criteria; 70 received SBRT, and 57 received SRS. Eighty-three patients were treated for non-small cell lung cancer, 7 patients for small cell lung cancer, and 35 patients for other cancers, with the most common one being melanoma. Fifty-three percent of patients received nivolumab, 29% pembrolizumab, 13% atezolizumab, 5% other. Twenty percent received immunotherapy before SBRT/SRS, 39% during SBRT/SRS, 41% after. Eighty-six patients had died by the time of the analysis; the median OS for the whole cohort was 9.7 months. Patients who had completed immunotherapy prior to SBRT/SRS had worse OS than those who received concurrent therapy or immunotherapy after SBRT/SRS, with a difference in median OS of 3.6 months vs. 13.0 months (p = 0.010) that was retained on multivariate analysis (p = 0.011). There was no significant difference in OS between patients receiving SRS vs. SBRT (p = 0.20), sex (p = 0.53), age >62 years (p = 0.76), or lung primary vs. others (p = 0.73) on univariate or multivariate analysis. When comparing before/concurrent to after/concurrent administration, there is a difference in survival with after/concurrent survival of 8.181 months and before survival of 13.010 months, but this was not significant (p = 0.25).

**Conclusions:**

OS appears to be worse in patients who complete immunotherapy prior to SBRT/SRS compared to those receiving it concurrently or after. The design of this retrospective review may be prone to lead time bias, although the difference in median survival is longer than the 6-month window before SBRT/SRS and could only account for part of this difference. Further analysis into causes of death and toxicity and prospective studies are needed to confirm the results of this analysis.

## Introduction

Cancer remains a leading cause of death in developed countries. One therapeutic technique that has yet to be optimized is the combination of local radiation therapy and immunotherapy, which is thought to have a synergistic effect (1–4). Since 1953, radiation therapy has been found to aid antitumor activity outside of the irradiated site in a phenomenon known as the abscopal effect (5–8). The exact mechanism behind the abscopal effect is unknown, but current research suggests that local radiation generates tumor-specific antigens that are processed and presented to T cells for systemic antitumor activity (2, 6, 8, 9). However, the inability to predict when the abscopal effect will happen had led to clinical limitations. In addition, the posited idea of antigen generation by radiation may be limited by modulators of the immune system including program cell death protein-1 (PD-1) and program cell death protein ligand-1 (PD-L1) (6, 8–10). For instance, Park et al. (10) showed that the abscopal effect induced by stereotactic ablative radiotherapy (SABR) was minimized by PD-1 in preclinical mouse models of melanoma and renal cell carcinoma. New immunotherapy medications that target immune modulators such as PD-1 and PD-L1 have led to a new interest in the abscopal effect with radiation. For instance, Smilowitz et al. (11) showed in 2016 that combining radiation therapy with immune checkpoint inhibitors improved outcomes in a mouse model of intracerebral melanoma. As such, it is thought that immune checkpoint inhibitors present a way to circumvent the immunosuppressive components that limit the influence of abscopal effects.

There remain a lot of unknowns about the relationship between immunotherapy and the abscopal effect. In particular, the optimal sequence of immunotherapy with radiation remains unknown (12). Specifically, is immunotherapy best to give before, concurrently, or after localized radiation? Various studies have indicated that the optimal sequence remains unknown (12).

To analyze the optimal sequence, we conducted a retrospective review at our institution of the best time to administer immunotherapy targeting PD-1 and PD-L1 with localized radiation. A preclinical study in 2014 suggested that concurrent delivery of radiation with PD-L1 antagonism was better than subsequent administration of immunotherapy (13). Initial experience at our hospital also suggested similar results with the use of PD-1 inhibitors and stereotactic body radiotherapy (SBRT) or stereotactic radiosurgery (SRS) (14). Our hypothesis was that administering immunotherapy concurrently with SBRT (i.e., a sandwiched approach) would produce the best improvement in overall survival (OS) compared to administering immunotherapy before SBRT/SRS or after SBRT/SRS.

## Materials and Methods

We conducted a single-center retrospective chart review for this study. The study was conducted with institutional review board (IRB) approval under UMC IRB 15-001726.

### Patient Selection

Patients were selected for review if they had received PD-1 or PD-L1 immunotherapy and SBRT/SRS within 6 months of each other from January 1, 2014, to December 31, 2019. This was initially established from an institutional lung database, but we expanded our data set to include cancers other than lung (14).

### Patient Data Collection

The selected patients’ charts were reviewed to collect the following information: age, gender, date of birth, date of cancer diagnosis, cancer pathology, cancer stage at diagnosis, any metastasis that existed at the time of immunotherapy administration, site of radiation treatment, type of radiation treatment (SBRT vs. SRS), date(s) of radiation treatment, fractionation or dose of radiation treatment, immunotherapy drug used, immunotherapy dates of treatment, the date it was decided to start immunotherapy, failure or progression following immunotherapy, if a patient was alive or deceased, and date last known alive or date of death. If the patient was still alive on December 31, 2019, this date was chosen as his or her last known alive/death date.

Patients were then classified into one of three categories as to when they received their immunotherapy relative to their radiation treatment: before radiation, after radiation, or sandwiched with radiation treatment. To encompass patients who may experience the abscopal effect, only patients who received immunotherapy concurrently, 6 months before, or 6 months after radiation treatment were included in the study. This time frame was based on discussions with multiple clinicians at our site and the time frame they would use to combine radiation with immunotherapy to induce the abscopal effect, although reports of the abscopal effect lasting up to 12 months have been reported (15). Some patients received multiple rounds of immunotherapy and radiation treatment during their cancer care between the 2014 and 2019 time frame. Since this study’s hypothesis focused on the sandwiched radiation to immunotherapy effect, if a patient received immunotherapy sandwiched with radiation, then this was the prioritized time frame used for this study. In such cases where a patient received multiple courses of immunotherapy concurrently with radiation, then the immunotherapy course closest to radiation was used. If a patient did not receive radiation and immunotherapy concurrently, then the most recent course of radiation/immunotherapy was used.

### Data Analysis

Our primary endpoint was OS as calculated from the end of SBRT/SRS to a patient’s last known alive date. Significance was defined as a p-value <0.05. All statistical analyses were performed using MedCalc Statistical Software (v20.013, Acacialaan 22, B-8400 Ostend Belgium). Kaplan–Meier survival analysis was conducted using the log-rank test. Multivariate analysis was performed using Cox multiple regression analysis.

## Results

### Patient Demographics

In total, we found 125 patients who fit our inclusion criteria, and the results are summarized in **Table 1**. There were 56% men and 44% women. Ninety (72%) patients had lung cancer with adenocarcinoma (47.2% patients), squamous cell carcinoma (19.2%), or small cell carcinoma (5.6%). Thirty-five (28%) patients had other histological cancers including 9 patients with melanoma (7.2%). About 55% received SBRT, and 45% received SRS. In this study, 54.4% of the patients received nivolumab, 31.2% received pembrolizumab, 12.8% received atezolizumab, and 0.8% received avelumab or durvalumab as immunotherapy.

**Table 1:** Patient demographics including sex, cancer type (divided into subclassifications for lung cancer), type of radiation therapy, timing of immunotherapy, and exact immunotherapy used.

DEMOGRAPHICS (n = 125)	
Men	70 (56%)
Women	55 (44%)
Lung cancer	90 (72.0%)
• Adenocarcinoma	59 (47.2%)
• Squamous cell	24 (19.2%)
• Small cell	7 (5.6%)
Non-lung cancer	35 (28.0%)
• Melanoma	9 (7.2%)
• Poorly differentiated	4 (3.2%)
• Head and neck	3 (2.4%)
• Neuroendocrine	2 (1.6%)
• Thymic	2 (1.6%)
• Other (1 case of each)	15 (16%)
**TYPE OF THERAPY**	
SBRT	68 (54.4%)
SRS	57 (45.6%)
	
**TIMING OF IMMUNOTHERAPY**	
Before	26 (20.8%)
After	51 (40.8%)
During	48 (38.4%)
	
**IMMUNOTHERAPY**	
Nivolumab (PD-1)	68 (54.4%)
Pembrolizumab (PD-1)	39 (31.2%)
Atezolizumab (PD-L1)	16 (12.8%)
Avelumab (PD-L1)	1 (0.8%)
Durvalumab (PD-L1)	1 (0.8%)

SBRT, Stereotactic Body Radiotherapy; SRS, Stereotactic Radiosurgery.

### Primary Endpoint

**Figure 1:**
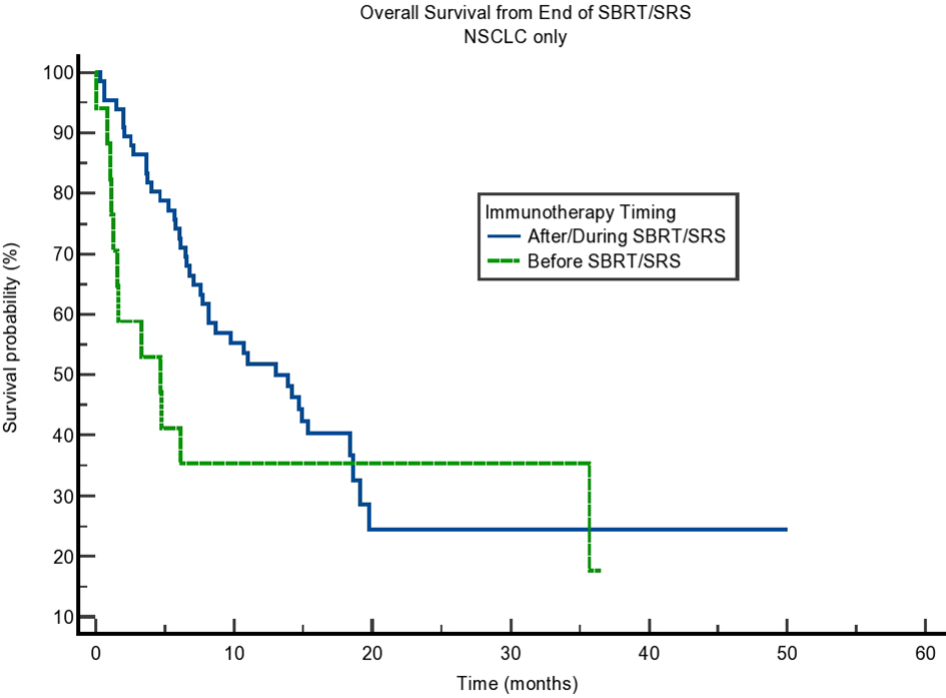
Kaplan–Meier curve showing overall survival (OS) from the end of stereotactic body radiotherapy (SBRT)/stereotactic radiosurgery (SRS). Comparing immunotherapy when given during/after SBRT/SRS compared to before SBRT/SRS. p = 0.014.

We calculated OS from the end of radiation therapy to the patient’s last known alive date or his/her death. We found that the OS of patients receiving immunotherapy before radiation was 3.285 months compared to 13.010 months for patients who received immunotherapy concurrently or after radiation (p = 0.014). The curve can be seen in **Figure 1** and results in **Table 2**. This difference in OS was maintained on multivariate analysis accounting for patient age (p = 0.76), sex (0.53), cancer type (lung vs. non-lung) (0.73), and modality of radiation (SBRT/SRS) (p = 0.20) as seen in **Table 3**. In comparison, the median OS from SBRT/SRS was not significantly different in other comparisons. For patients who received immunotherapy before/concurrently with radiation, OS was 8.181 months compared to 12.682 months for those patients who received immunotherapy after radiation (p = 0.33). For patients who received immunotherapy concurrently with radiation, OS was 13.010 months vs. 8.641 months for those who received it before or after radiation (p = 0.46).

**Table 2:** Median OS from SBRT in months as calculated from the end of radiation treatment.

Immunotherapy	Median OS from SBRT (months)	p-value
Before*	3.285	0.014
Concurrent/After*	13.010	
Before/Concurrent	8.181	0.33
After	12.682	
Concurrent	13.010	0.46
Before/After	8.641	

*Indicates significance. OS, Overall Survival; SBRT, Stereotactic Body Radiotherapy.

**Table 3:** Multivariate analysis results showing that immunotherapy timing remained significant when accounting for age, sex, type of cancer (lung vs. other), and type of radiation treatment.

Multivariate Analysis	p-value
Immunotherapy Timing (Before vs. During/After)	0.0
Age > or <62 years	0.76
Gender	0.53
Lung Cancer vs. Non lung cancer	0.73
SBRT vs. SRS	0.20

SBRT, Stereotactic Body Radiotherapy; SRS, Stereotactic Radiosurgery.

**Figure 2:**
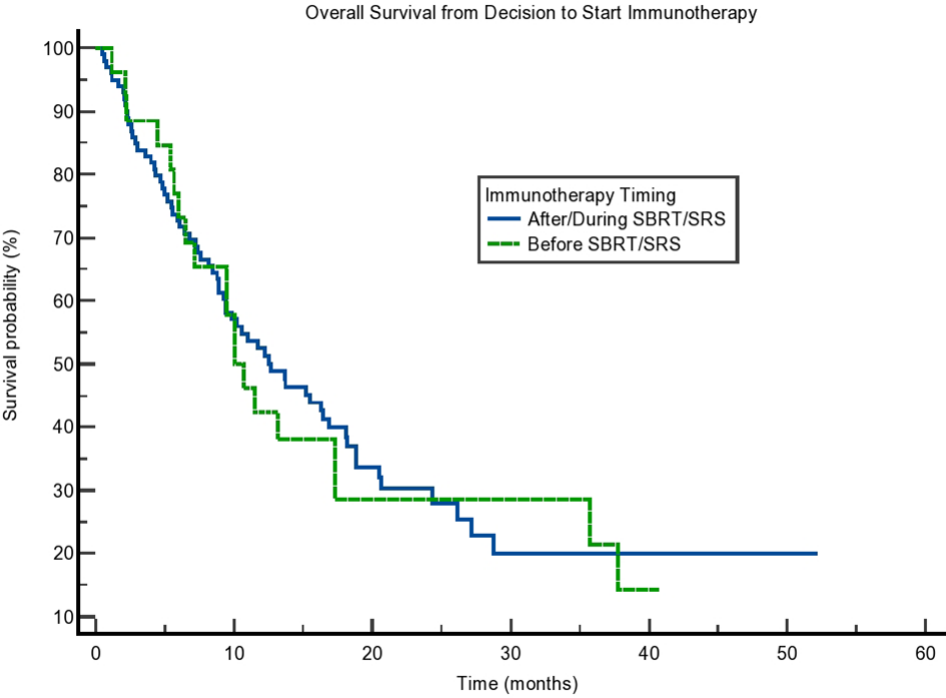
Kaplan–Meier curve showing overall survival (OS) from the end of treatment. OS separated based on immunotherapy given during/after stereotactic body radiotherapy (SBRT)/stereotactic radiosurgery (SRS) compared to before SBRT/SRS. p = 0.064.

**Figure 3:**
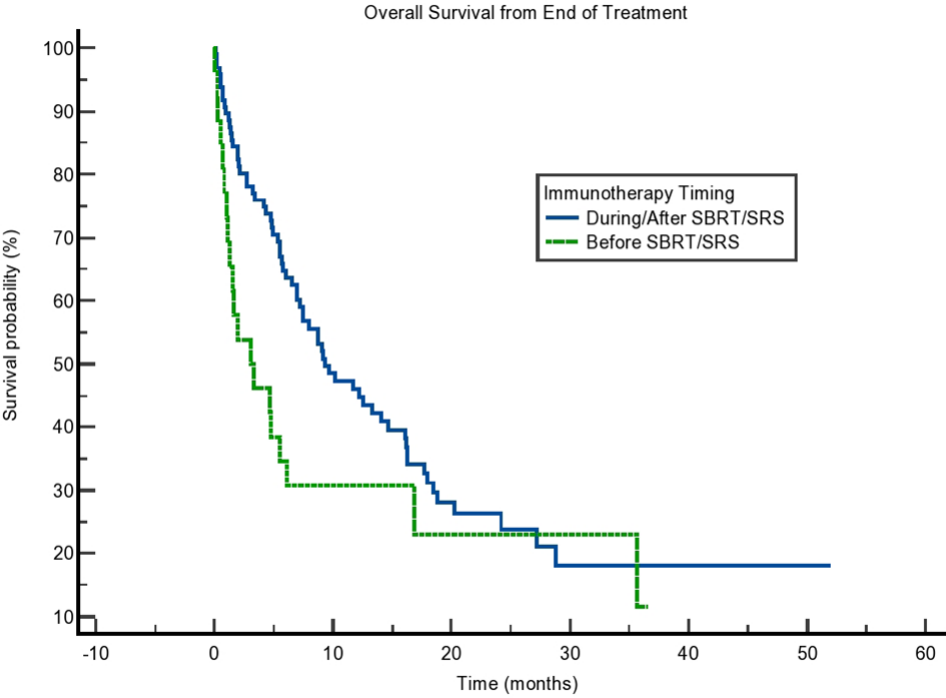
Kaplan–Meier curve showing overall survival (OS) from the decision to start immunotherapy. OS based on immunotherapy given during/after stereotactic body radiotherapy (SBRT)/stereotactic radiosurgery (SRS) compared to before SBRT/SRS. p = 0.81.

Due to the potential for lead time bias in our calculation, we also calculated OS starting from the end of all SBRT/immunotherapy treatment and starting from the decision to treat with immunotherapy. These results are noted in **Figures 2**, **3**, respectively. OS as calculated from the end of treatment of either radiation or immunotherapy was longer for those who received immunotherapy concurrently/after at 9.331 months compared to those who received immunotherapy before radiation at 3.088 months, but that result was not significant (p = 0.064). This difference in OS between these cohorts is not present when OS is calculated from the decision to start immunotherapy. Those who received immunotherapy before survived 10.02 months compared to 12.65 months for those who received immunotherapy concurrently or after radiation (p = 0.81). These can be seen in **Figure 3** and **Table 4**.

**Table 4:** Median OS as calculated from the end of treatment (immunotherapy or radiation) or decision to start immunotherapy.

Endpoint for OS	Immunotherapy Timing	Median OS from SBRT (months)	p-value
End of treatment	Before	3.088	0.064
	Concurrent/After	9.331	
Decision to start immunotherapy	Before	10.02	0.91
	Concurrent/After	12.65	

OS, Overall survival; SBRT, Stereotactic Body Radiotherapy.

We tested the difference between immunotherapy given before radiation vs. being given concurrently/after radiation at a shortened interval between immunotherapy and radiation of 3 months and 1 month, respectively. Within 3 months, the OS for patients who received immunotherapy before radiation was 1.971 months compared to 9.725 months for those who received it concurrently or after radiation. This difference was smaller at 1 month with an OS of 3.285 months for those who received immunotherapy before radiation compared to 9.593 months for those who received immunotherapy concurrently or after radiation. Of note, the differences in OS at 3 months (p = 0.17) and 1 month (p = 0.57) were not statistically significant.

**Figure 4:**
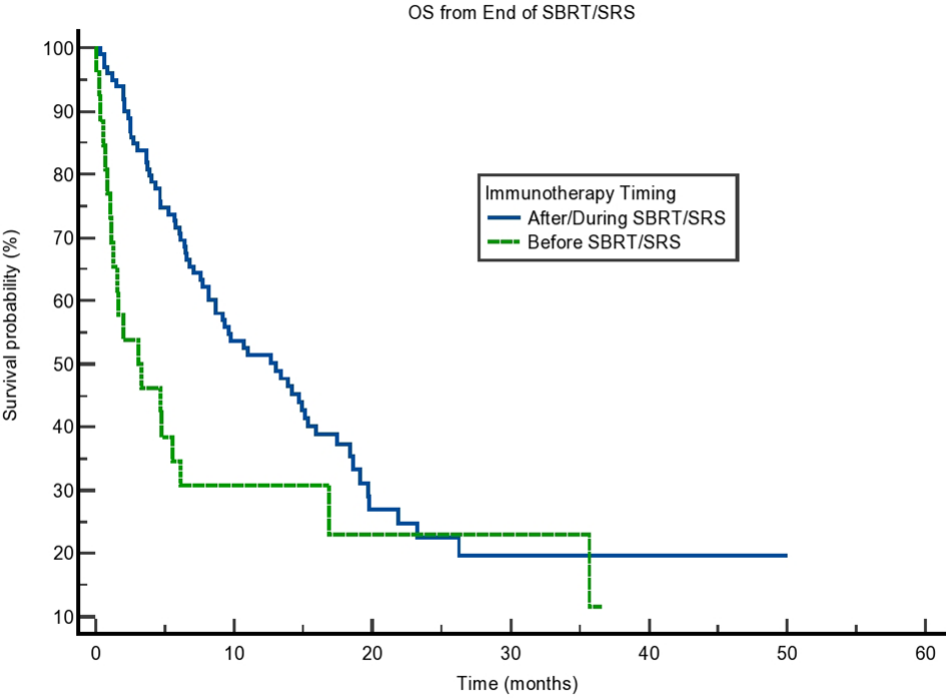
Kaplan–Meier curve showing overall survival (OS) from the end of stereotactic body radiotherapy (SBRT)/stereotactic radiosurgery (SRS) for non-small cell lung cancer (NSCLC) patients only. OS based on immunotherapy given during/after SBRT/SRS compared to before SBRT/SRS. p = 0.16.

We also calculated the difference in OS of those with non-small cell lung cancer (NSCLC), as this group comprised most of our population. Interestingly, although the difference in OS was present, it was not statistically significant (p = 0.16). The results can be seen in **Figure 4**. Finally, we tested SBRT/SRS comparisons of survival. While SBRT had a trend toward a difference in OS, there was a statistical difference in terms of SRS patients. This can be seen in **Table 5**. This result is intriguing and opens the possibility of studying SRS timing with immunotherapy in more detail.

**Table 5:** Median OS as calculated from the end of radiation treatment based on the type of radiation.

Type of Radiation	Immunotherapy Timing	Median OS from SBRT (months)	p-value
SBRT	Before	6.538	0.64
	Concurrent/After	12.583	
SRS	Before*	3.088	0.0098
	Concurrent/After*	9.593	

*Indicates significance. OS, Overall survival; SBRT, Stereotactic Body Radiotherapy; SRS, Stereotactic Radiosurgery.

## Discussion

Based on our analysis, we see that the OS was significantly greater for those patients receiving immunotherapy concurrently or after radiation compared to those who received immunotherapy prior to radiation. This contrasts with previous preliminary data we had that suggested that immunotherapy sandwiched between SBRT/SRS was more beneficial, but the data were more limited in that abstract (14). A recently published phase 2 trial also showed improvement for patients who received pembrolizumab after SBRT for NSCLC (16). However, results did not meet significance as defined by the trial and were influenced by patients with PD-L1-positive tumors. Therefore, the data on how to combine therapies are still inconclusive. With respect to OS based on the decision to start immunotherapy, our results were not significant. We suspect that this may also be confounded due to differing times between the decision to start immunotherapy and the patient actually receiving immunotherapy, but this is a valuable clinical decision point. As such, the applicability of our results to clinical decision-making remains limited.

Our data expand on existing knowledge about the optimal timing of radiation therapy and immunotherapy. Secondary review of the KEYNOTE-001 trial suggested that receiving pembrolizumab after radiation promoted greater progression-free survival and OS for NSCLC. This was for any amount of time prior to pembrolizumab treatment (17). A year later, a phase 1 trial that treated urothelial carcinoma with radiotherapy and pembrolizumab demonstrated that the sandwiched approach was more effective than immunotherapy after radiation (18). Interestingly, our results also show that either a sandwiched approach of immunotherapy and radiation or providing immunotherapy after radiation shows benefit.

Of note, there are several limitations to our study. As mentioned above, external application is limited clinically because there is no significant difference in OS in the timing of immunotherapy/radiation when analyzed from the decision to start immunotherapy. While the end of radiation is a usable point in a retrospective review, its use as a clinical decision point is not feasible.

Additionally, as a retrospective review, we attempt to limit inherent differences in the groups of selections. However, the OS of those patients who received immunotherapy prior to SBRT/SRS was significantly lower than those who received immunotherapy concurrently or after. This suggests a difference in the groups outside of the parameters chosen for inclusion and exclusion in the study.

Furthermore, our study has analyzed several different types of cancer in addition to combining SBRT and SRS, and this leads to concerns over its applicability. Overall, our results show the need for clinical trials with robust methodology that specifies sequencing of immunotherapy and radiation and does so for a particular cancer. Chemotherapy regimens are currently delineated for specific cancers, and the same approach should be applied when using immunotherapy and radiation.

Our results raise more questions about determining the optimal sequence for radiation and immunotherapy. There needs to be a wider collection of retrospective data combined with randomized trials to determine what sequence is best for what cancer. In addition to the timing of SBRT/SRS with immunotherapy, there is a need to optimize other portions of SBRT/SRS and immunotherapy administration including the optimal dose of radiation and fractionation. Overall, more data are needed to elucidate the optimal timing of immunotherapy with SBRT/SRS. There are several studies currently exploring this topic (2–4), and it is important that the full benefit of this combination is understood to improve clinical care.

## Data Availability Statement

The raw data supporting the conclusions of this article will be made available by the authors without undue reservation.

## Ethics Statement

The studies involving human participants were reviewed and approved by UMC IRB 15-001726. Written informed consent for participation was not required for this study in accordance with the national legislation and the institutional requirements.

## Author Contributions

AH, AJ, HA, PW, and NS aided in designing the study. SW and AH collected the data for the retrospective review. SW, AJ, and MP contributed to the statistical analysis and wrote the first draft of the article. All authors contributed to article revision and read and approved the submitted version.

## Conflict of Interest

Author PW was employed by Circulogene, Birmingham, AL, United States.

The remaining authors declare that the research was conducted in the absence of any commercial or financial relationships that could be construed as a potential conflict of interest.

## Publisher’s Note

All claims expressed in this article are solely those of the authors and do not necessarily represent those of their affiliated organizations, or those of the publisher, the editors and the reviewers. Any product that may be evaluated in this article, or claim that may be made by its manufacturer, is not guaranteed or endorsed by the publisher.
